# Antiphospholipid syndrome-associated nephropathy: Current concepts

**DOI:** 10.12861/jrip.2013.01

**Published:** 2013-03-01

**Authors:** Hamid Nasri

**Affiliations:** ^1^Department of Nephrology, Division of Nephropathology, Isfahan University of Medical Sciences, Isfahan, Iran

**Keywords:** Antiphospholipid antibodies, ISN/RPS2003 lupus nephropathy classification, Anti-phospholipid syndrome associated nephropathy

**Implication for health policy/practice/research/medical education:**

Renal pathologists and nephrologists should be aware of the morphologic characteristics of APS-nephropathy when they reviewbiopsies of lupus nephropathy patients, especially those with positive antiphospholipid antibodies.

Recently much attention was directed toward to find the morphologic lesions of antiphospholipid syndrome-associated nephropathy during the examination of renal biopsies of systemic lupus erythematosus patients, especially those with positive antiphospholipid antibodies (1). Recently Wu *et al*. conducted a study on, 341 patients with lupus nephritis, they found, 279 were diagnosed with single or multiple renal vascular lesions that included 253 with vascular immune complex deposits, 13 with non inflammatory necrotizing vasculopathy, 60 with thrombotic microangiopathy, 82 with atherosclerosis and 2 with true renal vasculitis. In this study they proposed to include of renal vascular lesions to the 2003 ISN/RPS system of lupus nephritis classification to improve renal outcome predictions (2). It is clear that the main etiologic factor of vasculopathy in systemic lupus erythematosus belongs to anti-phospholipid syndrome associated nephropathy (APS-nephropathy) which is also known as vaso-occlusive nephropathy (1-3). Morphologic lesions of this syndrome consist of acute lesions named thrombotic microangiopathy or chronic morphologic lesions mainly arteriosclerosis, fibrous intimal hyperplasia, fibrous occlusions of vessels, recanalized thrombi and focal cortical atrophy (2-4). It is well accepted that, morphologic lesions of APS-nephropathy aggravate the lupus nephropathy (3-6). Indeed, it is possible to suggest a distinct classification for APS-nephropathy, to emphasis more consideration to this disease and to avoid underrecognition of this syndrome (2-5). This classification may be used together with the ISN/RPS 2003 of lupus nephropathy in the same report. Hence renal pathologists should be aware of the morphologic characteristics of APS-nephropathy when they study kidney, biopsies of lupus erythematosus patients, especially those with positive antiphospholipid antibodies (4-8). It has become evident that most of the vascular lesions previously thought of as lupus vasculopathy, are now known to be due to APS-nephropathy (7-10). In this regard, Oxford classification for IgA nephropathy is a good example for morphologic lesions of APS-nephropathy. Thus in the cases of co-association of lupus nephritis and APS-nephropathy, this classification can be used together with lupus nephropathy classification, to avoid neglecting of APS-nephropathy.

There are some points, should explain more:

1- In contrast to the morphologic lesions of lupus nephritis, which is usually additives, pathologic features of APS-nephropathy was not proliferative. Indeed, in lupus nephropathy, pathologic lesions may evolve from class I to II, III, IV and in the case of failure to treatment, class VI lupus nephritis will come. While in APS-nephropathy, pathologic damage is mainly due to vaso-occlusion, affects glomeruli, vessels and tubulointerstitial area.
2- In case of proposing a classification like Oxford classification it is necessary to include the vascular lesions too.

However, the mostly important question is, which morphologic lesions are mostly important and should include into a proposing classification for APS-nephropathy. We propose to categorize the vascular lesions into acute (thrombotic microangiopathy) and chronic (fibrose intimal hyperplasia, thrombus), glomerular lesions (glomerular ballooning) and tubule-interstitial involvement (focal cortical atrophy, tubular thyroidization). In this regard we suggest more studies.

## Author’s contribution

HN is the single author of the manuscript

## Conflict of interests

The author declared no competing interests.

## Ethical considerations

Ethical issues (including plagiarism, data fabrication, double publication) have been completely observed by the author.

## Funding/Support

None.
